# Supplementary material for: Clinical and analytical validation of FoundationOne®CDx, a comprehensive genomic profiling assay for solid tumors
Source: PLoS One. 2022 Mar 16;17(3):e0264138. doi: 10.1371/journal.pone.0264138 (PMC8926248; doi:10.1371/journal.pone.0264138)
Supplement: S1 Appendix — (DOCX) [file pone.0264138.s001.docx]

**S1 Supplemental Appendix: Table S1−Table S40**

**Table S1. Abbreviations and acronyms**

| ACCE | Analytical validity, Clinical validity, Clinical utility, and Ethical, legal and social implications of genetic testing |
| --- | --- |
| ALK | Anaplastic lymphoma kinase |
| BICR | Blinded Independent Central Radiology Review |
| bp | base pair |
| BRAF | v-Raf murine sarcoma viral oncogene homolog B |
| BRCA | Breast cancer gene |
| BWA | Burrows-Wheeler Aligner |
| CAP | College of American Pathologists |
| CDx | Companion diagnostic |
| CGP | Comprehensive genomic profiling |
| CI | Confidence interval |
| CLIA | Clinical laboratory improvement amendments |
| CMS | Centers for Medicare & Medicaid Services |
| CN | Copy number |
| CNA | Copy number alteration |
| CR | Complete response |
| CRC | Colorectal cancer |
| CTA | Clinical trial assay |
| CUP | Carcinoma of unknown primary |
| dbSNP | Single Nucleotide Polymorphism Database |
| DOR | Duration of response |
| DV | Device validation |
| EGFR | Epidermal growth factor receptor |
| ERBB2 | Erb-B2 receptor tyrosine kinase 2 |
| evNGS | Externally validated next-generation sequencing |
| evWES | Externally validated whole exome sequencing |
| F1 LDT | FoundationOne® Laboratory Developed Test |
| F1CDx | FoundationOne®CDx |
| FDA | United States Food and Drug Administration |
| FFPE | Formalin-fixed, paraffin-embedded |
| FGFR2 | Fibroblast growth factor receptor 2 |
| FISH | Fluorescence in situ hybridization |
| GIST | Gastrointestinal stromal tumor |
| gLOH | Genomic loss of heterozygosity |
| gnomAD | Genome Aggregation Database |
| HR | Hazard ratio |
| HRD | Homologous recombination deficiency |
| HRR | Homologous recombination repair |
| HRRm | Homologous recombination repair mutation |
| ICPI | Immune checkpoint inhibitor |
| IHC | Immunohistochemistry |
| INDEL | Insertion/deletion |
| IRB | Institutional review board |
| KRAS | V-Ki-ras2 Kirsten rat sarcoma |
| LoB | Limit of blank |
| LoD | Limit of detection |
| LOH | Loss of heterozygosity |
| MAF | Mutant allele frequency |
| Mb | Mega base |
| mCRPC | Metastatic castration-resistant prostate cancer |
| MET | Mesenchymal-epithelial transition |
| MQ | Mapping quality |
| MSI | Microsatellite instability |
| MTB | Molecular Tumor Board |
| Mut | Mutation |
| N/A | Not applicable |
| NCCN | National Comprehensive Cancer Network |
| NCD | National coverage determination |
| ncRNA | Non-coding RNA |
| NGS | Next generation sequencing |
| NHA | Novel hormonal agent |
| NPA | Negative percent agreement |
| NSCLC | Non-small cell lung cancer |
| NTRK | Neurotrophic receptor tyrosine kinase |
| ORR | Objective response rate |
| OS | Overall survival |
| PCWG3 | Prostate Cancer Working Group 3 |
| PD-L1 | Programmed death-ligand 1 |
| PFS | Progression-free survival |
| PIK3CA | Phosphatidylinositol-4,5-bisphosphate 3-kinase catalytic subunit alpha |
| PMA | Premarket approval |
| PNS | Peripheral nervous system |
| PPA | Positive percent agreement |
| PR | Partial response |
| QC | Quality control |
| RCT | Randomized controlled trial |
| RECIST | Response Evaluation Criteria in Solid Tumors |
| rPFS | Radiological progression-free survival |
| RT-PCR | Reverse transcriptase-polymerase chain reaction |
| SGZ | Somatic-germline/zygosity |
| SNP | Single nucleotide polymorphism |
| sPMA | Supplemental premarket approval |
| SUB | Base substitution |
| SV | Short variant |
| TE | Therapeutic efficacy |
| TMB | Tumor mutational burden |
| TMB-H | Tumor mutational burden high |
| TTF | Time to treatment failure |
| VUS | Variant of unknown significance |
| WES | Whole exome sequencing |
| WGS | Whole genome sequencing |

**Table S2. Genes with full coding exonic regions included in F1CDx for the detection of SUBs, INDELs, rearrangements, and CNAs**

| *ABL1* | *BRAF* | *CDKN1A* | *EPHA3* | *FGFR4* | *IKZF1* | *MCL1* | *NKX2-1* | *PMS2* | *RNF43* | *TET2* |
| --- | --- | --- | --- | --- | --- | --- | --- | --- | --- | --- |
| *ACVR1B* | *BRCA1* | *CDKN1B* | *EPHB1* | *FH* | *INPP4B* | *MDM2* | *NOTCH1* | *POLD1* | *ROS1* | *TGFBR2* |
| *AKT1* | *BRCA2* | *CDKN2A* | *EPHB4* | *FLCN* | *IRF2* | *MDM4* | *NOTCH2* | *POLE* | *RPTOR* | *TIPARP* |
| *AKT2* | *BRD4* | *CDKN2B* | *ERBB2* | *FLT1* | *IRF4* | *MED12* | *NOTCH3* | *PPARG* | *SDHA* | *TNFAIP3* |
| *AKT3* | *BRIP1* | *CDKN2C* | *ERBB3* | *FLT3* | *IRS2* | *MEF2B* | *NPM1* | *PPP2R1A* | *SDHB* | *TNFRSF14* |
| *ALK* | *BTG1* | *CEBPA* | *ERBB4* | *FOXL2* | *JAK1* | *MEN1* | *NRAS* | *PPP2R2A* | *SDHC* | *TP53* |
| *ALOX12B* | *BTG2* | *CHEK1* | *ERCC4* | *FUBP1* | *JAK2* | *MERTK* | *NT5C2* | *PRDM1* | *SDHD* | *TSC1* |
| *AMER1* | *BTK* | *CHEK2* | *ERG* | *GABRA6* | *JAK3* | *MET* | *NTRK1* | *PRKAR1A* | *SETD2* | *TSC2* |
| *APC* | *C11orf30* | *CIC* | *ERRFI1* | *GATA3* | *JUN* | *MITF* | *NTRK2* | *PRKCI* | *SF3B1* | *TYRO3* |
| *AR* | *CALR* | *CREBBP* | *ESR1* | *GATA4* | *KDM5A* | *MKNK1* | *NTRK3* | *PTCH1* | *SGK1* | *U2AF1* |
| *ARAF* | *CARD11* | *CRKL* | *EZH2* | *GATA6* | *KDM5C* | *MLH1* | *P2RY8* | *PTEN* | *SMAD2* | *VEGFA* |
| *ARFRP1* | *CASP8* | *CSF1R* | *FAM46C* | *GID4*  *(C17orf39)* | *KDM6A* | *MPL* | *PALB2* | *PTPN11* | *SMAD4* | *VHL* |
| *ARID1A* | *CBFB* | *CSF3R* | *FANCA* | *GNA11* | *KDR* | *MRE11A* | *PARK2* | *PTPRO* | *SMARCA4* | *WHSC1* |
| *ASXL1* | *CBL* | *CTCF* | *FANCC* | *GNA13* | *KEAP1* | *MSH2* | *PARP1* | *QKI* | *SMARCB1* | *WHSC1L1* |
| *ATM* | *CCND1* | *CTNNA1* | *FANCG* | *GNAQ* | *KEL* | *MSH3* | *PARP2* | *RAC1* | *SMO* | *WT1* |
| *ATR* | *CCND2* | *CTNNB1* | *FANCL* | *GNAS* | *KIT* | *MSH6* | *PARP3* | *RAD21* | *SNCAIP* | *XPO1* |
| *ATRX* | *CCND3* | *CUL3* | *FAS* | *GRM3* | *KLHL6* | *MST1R* | *PAX5* | *RAD51* | *SOCS1* | *XRCC2* |
| *AURKA* | *CCNE1* | *CUL4A* | *FBXW7* | *GSK3B* | *KMT2A*  *(MLL)* | *MTAP* | *PBRM1* | *RAD51B* | *SOX2* | *ZNF217* |
| *AURKB* | *CD22* | *CXCR4* | *FGF10* | *H3F3A* | *KMT2D*  *(MLL2)* | *MTOR* | *PDCD1* | *RAD51C* | *SOX9* | *ZNF703* |
| *AXIN1* | *CD274* | *CYP17A1* | *FGF12* | *HDAC1* | *KRAS* | *MUTYH* | *PDCD1LG2* | *RAD51D* | *SPEN* |  |
| *AXL* | *CD70* | *DAXX* | *FGF14* | *HGF* | *LTK* | *MYC* | *PDGFRA* | *RAD52* | *SPOP* |  |
| *BAP1* | *CD79A* | *DDR1* | *FGF19* | *HNF1A* | *LYN* | *MYCL* | *PDGFRB* | *RAD54L* | *SRC* |  |
| *BARD1* | *CD79B* | *DDR2* | *FGF23* | *HRAS* | *MAF* | *MYCN* | *PDK1* | *RAF1* | *STAG2* |  |
| *BCL2* | *CDC73* | *DIS3* | *FGF3* | *HSD3B1* | *MAP2K1* | *MYD88* | *PIK3C2B* | *RARA* | *STAT3* |  |
| *BCL2L1* | *CDH1* | *DNMT3A* | *FGF4* | *ID3* | *MAP2K2* | *NBN* | *PIK3C2G* | *RB1* | *STK11* |  |
| *BCL2L2* | *CDK12* | *DOT1L* | *FGF6* | *IDH1* | *MAP2K4* | *NF1* | *PIK3CA* | *RBM10* | *SUFU* |  |
| *BCL6* | *CDK4* | *EED* | *FGFR1* | *IDH2* | *MAP3K1* | *NF2* | *PIK3CB* | *REL* | *SYK* |  |
| *BCOR* | *CDK6* | *EGFR* | *FGFR2* | *IGF1R* | *MAP3K13* | *NFE2L2* | *PIK3R1* | *RET* | *TBX3* |  |
| *BCORL1* | *CDK8* | *EP300* | *FGFR3* | *IKBKE* | *MAPK1* | *NFKBIA* | *PIM1* | *RICTOR* | *TEK* |  |

**Table S3. Genes with select intronic regions for the detection of additional gene rearrangements, one with 3'UTR, one gene with a promoter region, and one ncRNA gene**

| *ALK*  *introns 18, 19* | *BRCA1*  *introns 2, 7, 8, 12, 16, 19, 20* | *ETV4*  *intron 8* | EZR  *introns 9- 11* | *KIT*  *intron 16* | *MYC*  *intron 1* | *NUTM1*  *intron 1* | *RET*  *introns 7-11* | *SLC34A2*  *intron 4* |
| --- | --- | --- | --- | --- | --- | --- | --- | --- |
| *BCL2*  *3’UTR* | *BRCA2*  *intron 2* | *ETV5*  *introns 6, 7* | *FGFR1*  *intron 1, 5, 17* | *KMT2A (MLL)*  *introns 6-11* | *NOTCH2*  *intron 26* | *PDGFRA*  *introns 7, 9, 11* | *ROS1*  *introns 31-35* | *TERC*  *ncRNA* |
| *BCR*  *introns 8, 13, 14* | *CD74*  *introns 6- 8* | *ETV6*  *introns 5, 6** | *FGFR2*  *intron 1, 17* | *MSH2*  *intron 5* | *NTRK1*  *introns 8-11* | *RAF1*  *introns 4-8* | *RSPO2*  *intron 1* | *TERT*  Promoter |
| *BRAF*  *introns 7- 10* | *EGFR*  *introns 7, 15, 24-27* | *EWSR1 introns 7-13* | *FGFR3*  *intron 17* | *MYB*  *intron 14* | *NTRK2*  *Intron 12* | *RARA*  *intron 2* | *SDC4*  *intron 2* | *TMPRSS2 introns 1- 3* |

ncRNA = non-coding RNA; UTR = untranslated region.

*ETV6 is a common rearrangement partner for NTRK3.

**Table S4. Concordance table with CCD1, CCD2 and F1CDx results with eligible samples for *EGFR* Exon 19del/L858R**

|  | **CCD1+** | | | | **CCD1-** | | | |
| --- | --- | --- | --- | --- | --- | --- | --- | --- |
|  | **CCD2+** | **CCD2-** | **CCD2 missing** | **Total** | **CCD2+** | **CCD2-** | **CCD2 missing** | **Total** |
| F1CDx+ | 106 | 0 | 0 | 106 | 1 | 1^a^ | 0 | 2 |
| F1CDx- | 2^b^ | 1 | 0 | 3 | 3 | 153 | 0 | 153 |
| F1CDx missing | 3 | 0 | 0 | 3 | 1 | 9 | 2 | 12 |
| Total | 111 | 1 | 0 | 112 | 5 | 163 | 2 | 170 |

CCD1 = replicate 1 of the cobas *EGFR* mutation test; CCD2 = replicate 2 of the cobas *EGFR* mutation test; F1CDx = FoundationOne^®^CDx.

^a^ The sample noted here was the only sample where both replicates of the cobas® v2 *EGFR* mutation test reported negative results but F1CDx reported positive for L858R with AF 33%. Upon further review, F1CDx identified a second somatic mutation in-cis (on same allele) as that of L858R with identical AF only 17bp downstream: *EGFR* A864P. Therefore, it is suspected that this second mutation interfered with the allele-specific PCR primers of cobas® v2 *EGFR* mutation test, and thus L858R went undetected.

^b^ The samples noted here were reported as positive for both replicates of cobas® v2 EGFR assay for Exon 19del, but negative by F1CDx. For one sample, F1CDx detected the exon19 deletion, but incorrectly annotated the variant as 2 frameshift mutations. This would have been corrected by manual curation review, which was not part of this concordance study. For the second sample, F1CDx identified an 18bp exon 19 insertion event, with protein effect K745_E746insIPVAIK. As cobas® v2 EGFR assay is not designed to detect insertion events at exon 19, this result may indicate an error by cobas® v2 *EGFR* mutation test.

**Table S5. PPA and NPA for *EGFR* exon 19del/L858R**

|  | **Unadjusted for prevalence** | **Adjusted for prevalence (22.1%)** |
| --- | --- | --- |
| PPA_C1C2_ | 99.1% | 99.1% |
| PPA_C1F_ | 97.2% | 97.2% |
| NPA_C1C2_ | 97.5% | 97.5% |
| NPA_C1F_ | 98.7% | 98.7% |
| PPA_C2C1_ | 96.4% | 91.7% |
| PPA_C2F_ | 95.5% | 92.1% |
| NPA_C2C1_ | 99.4% | 99.7% |
| NPA_C2F_ | 99.4% | 99.4% |

CCD1 = replicate 1 of the cobas *EGFR* mutation test; CCD2 = replicate 2 of the cobas *EGFR* mutation test; NPA = negative percent agreement; PPA = positive percent agreement.

PPA_C1C2_ is the PPA between CCD1 and CCD2 conditional on CCD1.

PPA_C1F_ is the PPA between CCD1 and F1CDx conditional on CCD1.

PPA_C2C1_ is the PPA between CCD1 and CCD2 conditional on CCD2.

PPA_C2F_ is the PPA between CCD2 and F1CDx conditional on CCD2.

NPA_C1C2_ is the NPA between CCD1 and CCD2 conditional on CCD1.

NPA_C1F_ is the NPA between CCD1 and F1CDx conditional on CCD1.

NPA_C2C1_ is the NPA between CCD1 and CCD2 conditional on CCD2.

NPA_C2F_ is the NPA between CCD2 and F1CDx conditional on CCD2.

**Table S6. Point estimate and 95% one-sided upper bounds of ζ_PPA1_, ζ_NPA1_, ζ_PPA2_, and ζ_NPA2_ for *EGFR* exon 19del/L858R**

|  | **Mean** | **95% one-sided upper CI** |
| --- | --- | --- |
| ζ_PPA1_ | 1.9% | 4.6% |
| ζ_NPA1_ | -1.3% | 0.6% |
| ζ_PPA2_ | -0.3% | 3.4% |
| ζ_NPA2_ | 0.4% | 1.7% |

CI = confidence interval; NPA = negative percent agreement; PPA = positive percent agreement.

ζ_PPA1_=( PPA_C1C2_- PPA_C1F_); ζ_PPA2_=( PPA_C2C1_- PPA_C2F_); ζ_NPA1_=( NPA_C1C2_- NPA_C1F_); ζ_NPA2_=( NPA_C2C1_- NPA_C2F_)

**Table S7. Concordance table with CCD1, CCD2 and F1CDx results with eligible samples for *EGFR* T790M**

|  | **CCD1+** | | | | **CCD1-** | | | |
| --- | --- | --- | --- | --- | --- | --- | --- | --- |
|  | **CCD2+** | **CCD2-** | **CCD2 missing** | **Total** | **CCD2+** | **CCD2-** | **CCD2 missing** | **Total** |
| F1CDx+ | 87 | 19 | 1 | 107 | 8 | 15 | 0 | 23 |
| F1CDx- | 1 | 4 | 0 | 5 | 0 | 93 | 2 | 95 |
| F1CDx missing | 21 | 4 | 8 | 33 | 1 | 37 | 11 | 49 |
| Total | 109 | 27 | 9 | 145 | 9 | 145 | 13 | 167 |

CCD1 = replicate 1 of the cobas *EGFR* mutation test (v1 or v2); CCD2= replicate 2 of the cobas *EGFR* mutation test (v2); F1CDx = FoundationOne^®^CDx

**Table S8. PPA and NPA for *EGFR* T790M**

|  | **Unadjusted for prevalence** | **Adjusted for prevalence (62.5%)** |
| --- | --- | --- |
| PPA_C1C2_ | 79.3% | 79.3% |
| PPA_C1F_ | 95.5% | 95.5% |
| NPA_C1C2_ | 93.1% | 93.1% |
| NPA_C1F_ | 80.2% | 80.2% |
| PPA_C2C1_ | 91.7% | 95.0% |
| PPA_C2F_ | 99.0% | 98.9% |
| NPA_C2C1_ | 82.4% | 72.9% |
| NPA_C2F_ | 74.0% | 67.5% |

CCD1 = replicate 1 of the cobas *EGFR* mutation test; CCD2 = replicate 2 of the cobas *EGFR* mutation test; NPA = negative percent agreement; PPA = positive percent agreement.

PPA_C1C2_ is the PPA between CCD1 and CCD2 conditional on CCD1.

PPA_C1F_ is the PPA between CCD1 and F1CDx conditional on CCD1.

PPA_C2C1_ is the PPA between CCD1 and CCD2 conditional on CCD2.

PPA_C2F_ is the PPA between CCD2 and F1CDx conditional on CCD2.

NPA_C1C2_ is the NPA between CCD1 and CCD2 conditional on CCD1.

NPA_C1F_ is the NPA between CCD1 and F1CDx conditional on CCD1.

NPA_C2C1_ is the NPA between CCD1 and CCD2 conditional on CCD2.

NPA_C2F_ is the NPA between CCD2 and F1CDx conditional on CCD2.

**Table S9. Point estimate and 95% one-sided upper bounds of ζ_PPA1_, ζ_NPA1_, ζ_PPA2_, and ζ_NPA2_ for *EGFR* T790M**

|  | **Mean** | **95% one-sided upper CI** |
| --- | --- | --- |
| ζ_PPA1_ | -16.2% | -9.9% |
| ζ_NPA1_ | 12.9% | 18.1% |
| ζ_PPA2_ | -3.8% | -0.6% |
| ζ_NPA2_ | 5.5% | 11.2% |

CI = confidence interval; NPA = negative percent agreement; PPA = positive percent agreement.

ζ_PPA1_=( PPA_C1C2_- PPA_C1F_); ζ_PPA2_=( PPA_C2C1_- PPA_C2F_); ζ_NPA1_=( NPA_C1C2_- NPA_C1F_); ζ_NPA2_=( NPA_C2C1_- NPA_C2F_)

**Table S10. Concordance with CCD1, CCD2 and F1CDx results with eligible samples for *ALK* rearrangements**

|  | **CCD1+** | | | | **CCD1-** | | | |
| --- | --- | --- | --- | --- | --- | --- | --- | --- |
|  | **CCD2+** | **CCD2-** | **CCD2 missing** | **Total** | **CCD2+** | **CCD2-** | **CCD2 missing** | **Total** |
| F1CDx+ | 78 | 1 | 9 | 88 | 3 | 0 | 0 | 3 |
| F1CDx- | 6^a^ | 7 | 3 | 16 | 5 | 75 | 1 | 81 |
| F1CDx missing | 19 | 3 | 8 | 30 | 0 | 30 | 25 | 55 |
| Total | 103 | 11 | 20 | 134 | 8 | 105 | 26 | 139 |

CCD1 = Ventana *ALK* (D5F3) CDx Assay; CCD2 = *ALK* Break-Apart FISH Probe Kit; F1CDx = FoundationOne^®^CDx.

^a^ Two samples harbored *ALK* rearrangements that were detected by F1CDx but were classified as negative based on the study protocol.

**Table S11. PPA and NPA for *ALK* rearrangements**

|  | **Unadjusted for prevalence** | **Adjusted for prevalence (16.5%)** |
| --- | --- | --- |
| PPA_C1C2_ | 91.3% | 91.3% |
| PPA_C1F_ | 85.9% | 85.9% |
| NPA_C1C2_ | 90.4% | 90.4% |
| NPA_C1F_ | 96.4% | 96.4% |
| PPA_C2C1_ | 91.3% | 65.2% |
| PPA_C2F_ | 88.0% | 73.6% |
| NPA_C2C1_ | 90.4% | 98.1% |
| NPA_C2F_ | 98.8% | 99.8% |

CCD1 = Ventana *ALK* (D5F3) CDx Assay; CCD2 = *ALK* Break-Apart FISH Probe Kit; NPA = negative percent agreement; PPA = positive percent agreement.

PPA_C1C2_ is the PPA between CCD1 and CCD2 conditional on CCD1.

PPA_C1F_ is the PPA between CCD1 and F1CDx conditional on CCD1.

PPA_C2C1_ is the PPA between CCD1 and CCD2 conditional on CCD2.

PPA_C2F_ is the PPA between CCD2 and F1CDx conditional on CCD2.

NPA_C1C2_ is the NPA between CCD1 and CCD2 conditional on CCD1.

NPA_C1F_ is the NPA between CCD1 and F1CDx conditional on CCD1.

NPA_C2C1_ is the NPA between CCD1 and CCD2 conditional on CCD2.

NPA_C2F_ is the NPA between CCD2 and F1CDx conditional on CCD2.

**Table S12. Point estimate and 95% one-sided upper bounds of ζ_PPA1_, ζ_NPA1_, ζ_PPA2_, and ζ_NPA2_ for *ALK* rearrangements**

|  | **Mean** | **95% one-sided upper CI** |
| --- | --- | --- |
| ζ_PPA1_ | 5.43% | 9.78% |
| ζ_NPA1_ | -6.02% | -2.40% |
| ζ_PPA2_ | -8.40% | 3.37% |
| ζ_NPA2_ | -1.63% | -0.71% |

CI = confidence interval; NPA = negative percent agreement; PPA = positive percent agreement.

ζ_PPA1_=( PPA_C1C2_- PPA_C1F_); ζ_PPA2_=( PPA_C2C1_- PPA_C2F_); ζ_NPA1_=( NPA_C1C2_- NPA_C1F_); ζ_NPA2_=( NPA_C2C1_- NPA_C2F_)

**Table S13. Concordance table with CCD1, CCD2 and F1CDx results with eligible samples for *ERBB2* (HER2) amplification**

|  | **CCD1+** | | | **CCD1-** | | |
| --- | --- | --- | --- | --- | --- | --- |
|  | **CCD2+** | **CCD2-** | **Total** | **CCD2+** | **CCD2-** | **Total** |
| F1CDx+ | 101 | 2 | 103 | 3 | 3 | 6 |
| F1CDx- | 12 | 10 | 22 | 6 | 180 | 186 |
| Total | 113 | 12 | 125 | 9 | 183 | 192 |

CCD1 = replicate 1 of the HER2 FISH PharmDx^®^ Kit; CCD2 = replicate 2 of the HER2 FISH PharmDx^®^ Kit; F1CDx = FoundationOne^®^CDx.

**Table S14. PPA and NPA for *ERBB2* (HER2) amplification**

|  | **Unadjusted for prevalence** | **Adjusted for prevalence (62.5%)** |
| --- | --- | --- |
| PPA_C1C2_ | 90.4% | 90.4% |
| PPA_C1F_ | 82.4% | 82.4% |
| NPA_C1C2_ | 95.3% | 95.3% |
| NPA_C1F_ | 96.9% | 96.9% |
| PPA_C2C1_ | 92.6% | 80.4% |
| PPA_C2F_ | 85.2% | 78.4% |
| NPA_C2C1_ | 93.8% | 97.9% |
| NPA_C2F_ | 97.4% | 98.0% |

CCD1 = replicate 1 of the cobas *EGFR* mutation test; CCD2 = replicate 2 of the cobas *EGFR* mutation test; NPA = negative percent agreement; PPA = positive percent agreement.

PPA_C1C2_ is the PPA between CCD1 and CCD2 conditional on CCD1.

PPA_C1F_ is the PPA between CCD1 and F1CDx conditional on CCD1.

PPA_C2C1_ is the PPA between CCD1 and CCD2 conditional on CCD2.

PPA_C2F_ is the PPA between CCD2 and F1CDx conditional on CCD2.

NPA_C1C2_ is the NPA between CCD1 and CCD2 conditional on CCD1.

NPA_C1F_ is the NPA between CCD1 and F1CDx conditional on CCD1.

NPA_C2C1_ is the NPA between CCD1 and CCD2 conditional on CCD2.

NPA_C2F_ is the NPA between CCD2 and F1CDx conditional on CCD2.

**Table S15. Point estimate and 95% one-sided upper bounds of ζ_PPA1_, ζ_NPA1_, ζ_PPA2_, and ζ_NPA2_ for *ERBB2* (HER2) amplification**

|  | **Point estimate** | **95% one-sided upper CI** |
| --- | --- | --- |
| ζ_PPA1_ | 8.0% | 12.8% |
| ζ_NPA1_ | -1.56% | 1.0% |
| ζ_PPA2_ | 1.99% | 9.3% |
| ζ_NPA2_ | -0.14% | 1.7% |

CI = confidence interval; NPA = negative percent agreement; PPA = positive percent agreement.

ζ_PPA1_=( PPA_C1C2_- PPA_C1F_); ζ_PPA2_=( PPA_C2C1_- PPA_C2F_); ζ_NPA1_=( NPA_C1C2_- NPA_C1F_); ζ_NPA2_=( NPA_C2C1_- NPA_C2F_)

**Table S16. Concordance table with CCD1, CCD2 and F1CDx results with eligible samples for *KRAS* alterations**

|  | **CCD1+** | | | | **CCD1-** | | | |
| --- | --- | --- | --- | --- | --- | --- | --- | --- |
|  | **CCD2+** | **CCD2-** | **CCD2 missing** | **Total** | **CCD2+** | **CCD2-** | **CCD2 missing** | **Total** |
| F1CDx+ | 173 | 0 | 2 | 175 | 0 | 0 | 0 | 0 |
| F1CDx- | 0 | 2 | 0 | 0 | 1 | 154 | 7 | 162 |
| F1CDx missing | 0 | 0 | 0 | 0 | 0 | 3 | 0 | 3 |
| Total | 173 | 2 | 2 | 177 | 1 | 157 | 7 | 165 |

CCD1 = replicate 1 of the therascreen *KRAS* RGQ PCR Kit; CCD2 = replicate 2 of the therascreen *KRAS* RGQ PCR Kit; F1CDx = FoundationOne^®^CDx

**Table S17. PPA and NPA for *KRAS* alterations**

|  | **Unadjusted for prevalence** | **Adjusted for prevalence for cetuximab (35.6%)** | **Adjusted for prevalence**  **for panitumumab (40%)** |
| --- | --- | --- | --- |
| PPA_C1C2_ | 98.9% | 98.9% | 98.9% |
| PPA_C1F_ | 98.9% | 98.9% | 98.9% |
| NPA_C1C2_ | 99.4% | 99.4% | 99.4% |
| NPA_C1F_ | 100.0% | 100.0% | 100.0% |
| PPA_C2C1_ | 99.4% | 98.8% | 99.0% |
| PPA_C2F_ | 99.4% | 98.8% | 99.0% |
| NPA_C2C1_ | 98.7% | 99.4% | 99.2% |
| NPA_C2F_ | 100.0% | 100.0% | 100.0% |

CCD1 = replicate 1 of the therascreen *KRAS* RGQ PCR Kit; CCD2 = replicate 2 of the therascreen *KRAS* RGQ PCR Kit; F1CDx = FoundationOne^®^CDx; NPA = negative percent agreement; PPA = positive percent agreement.

PPA_C1C2_ is the PPA between CCD1 and CCD2 conditional on CCD1.

PPA_C1F_ is the PPA between CCD1 and F1CDx conditional on CCD1.

PPA_C2C1_ is the PPA between CCD1 and CCD2 conditional on CCD2.

PPA_C2F_ is the PPA between CCD2 and F1CDx conditional on CCD2.

NPA_C1C2_ is the NPA between CCD1 and CCD2 conditional on CCD1.

NPA_C1F_ is the NPA between CCD1 and F1CDx conditional on CCD1.

NPA_C2C1_ is the NPA between CCD1 and CCD2 conditional on CCD2.

NPA_C2F_ is the NPA between CCD2 and F1CDx conditional on CCD2.

**Table S18. Point estimate and 95% one-sided upper bounds of ζ_PPA1_, ζ_NPA1_, ζ_PPA2_, and ζ_NPA2_ for *KRAS* alterations**

|  | **Point estimate (cetuximab)** | **95% one-sided upper CI (cetuximab)** | **Point estimate (panitumumab)** | **95% one-sided upper CI (panitumumab)** |
| --- | --- | --- | --- | --- |
| ζ_PPA1_ | 0.0% | 0.0% | 0.0% | 0.0% |
| ζ_NPA1_ | -0.7% | 0.0% | -0.7% | 0.0% |
| ζ_PPA2_ | 0.0% | 0.0% | 0.0% | 0.0% |
| ζ_NPA2_ | -0.6% | 0.0% | -0.8% | 0.0% |

CI = confidence interval; NPA = negative percent agreement; PPA = positive percent agreement.

ζ_PPA1_=( PPA_C1C2_- PPA_C1F_); ζ_PPA2_=( PPA_C2C1_- PPA_C2F_); ζ_NPA1_=( NPA_C1C2_- NPA_C1F_); ζ_NPA2_=( NPA_C2C1_- NPA_C2F_)

**Table S19. Concordance table with CCD1, CCD2 and F1CDx results with eligible samples for *BRAF* V600 mutations**

|  | **CCD1+** | | | **CCD1-** | | | |
| --- | --- | --- | --- | --- | --- | --- | --- |
|  | **CCD2+** | **CCD2-** | **Total** | **CCD2+** | **CCD2-** | **Total** |  |
| F1CDx+ | 166 | 0 | 166 | 3 | 14 | 17 |  |
| F1CDx- | 1 | 0 | 1 | 0 | 121 | 121 |  |
| Total | 167 | 0 | 167 | 3 | 135 | 138 |  |

CCD1 = replicate 1 of the cobas *BRAF* V600 mutation test; CCD2 = replicate 2 of the cobas *BRAF* V600 mutation test; F1CDx = FoundationOne^®^CDx.

**Table S20. PPA and NPA for *BRAF* V600 mutations**

|  | **Unadjusted for prevalence** | **Adjusted for prevalence (33.8%)** |
| --- | --- | --- |
| PPA_C1C2_ | 100.0% | 100.0% |
| PPA_C1F_ | 99.4% | 99.4% |
| NPA_C1C2_ | 97.8% | 97.8% |
| NPA_C1F_ | 87.7% | 87.7% |
| PPA_C2C1_ | 98.2% | 95.9% |
| PPA_C2F_ | 99.4% | 99.4% |
| NPA_C2C1_ | 100.0% | 100.0% |
| NPA_C2F_ | 89.6% | 89.6% |

CCD1 = replicate 1 of the cobas *BRAF* V600 mutation test; CCD2 = replicate 2 of the cobas *BRAF* V600 mutation test; NPA = negative percent agreement; PPA = positive percent agreement.

PPA_C1C2_ is the PPA between CCD1 and CCD2 conditional on CCD1.

PPA_C1F_ is the PPA between CCD1 and F1CDx conditional on CCD1.

PPA_C2C1_ is the PPA between CCD1 and CCD2 conditional on CCD2.

PPA_C2F_ is the PPA between CCD2 and F1CDx conditional on CCD2.

NPA_C1C2_ is the NPA between CCD1 and CCD2 conditional on CCD1.

NPA_C1F_ is the NPA between CCD1 and F1CDx conditional on CCD1.

NPA_C2C1_ is the NPA between CCD1 and CCD2 conditional on CCD2.

NPA_C2F_ is the NPA between CCD2 and F1CDx conditional on CCD2.

**Table S21. Point estimate and 95% one-sided upper bounds of ζ_PPA1_, ζ_NPA1_, ζ_PPA2_, and ζ_NPA2_ for *BRAF* V600 mutations**

|  | **Point estimate** | **95% one-sided upper CI** |
| --- | --- | --- |
| ζ_PPA1_ | 0.60% | 1.80% |
| ζ_NPA1_ | 10.14% | 14.49% |
| ζ_PPA2_ | -3.50% | 0.00% |
| ζ_NPA2_ | 10.37% | 14.90% |

CI = confidence interval; NPA = negative percent agreement; PPA = positive percent agreement.

ζ_PPA1_=( PPA_C1C2_- PPA_C1F_); ζ_PPA2_=( PPA_C2C1_- PPA_C2F_); ζ_NPA1_=( NPA_C1C2_- NPA_C1F_); ζ_NPA2_=( NPA_C2C1_- NPA_C2F_)

**Table S22. Concordance table with CCD1, CCD2 and F1CDx results with eligible samples for *BRAF* V600 dinucleotide samples**

|  | **THxID+** | **THxID-** | **THxID missing** | **Total** |
| --- | --- | --- | --- | --- |
| F1CDx+ | 26 | 0 | 3 | 29 |
| F1CDx- | 1 | 24 | 4 | 29 |
| Total | 27 | 24 | 7 | 58 |

F1CDx = FoundationOne^®^CDx; THxID = THxID *BRAF* kit.

**Table S23. Concordance table comparing *NTRK* fusion detection results between the F1CDx and the local CTA**

|  | **Local CTA result** | | |
| --- | --- | --- | --- |
| **F1CDx result** | **Positive** | **Negative** | **Total** |
| **Positive** | 37 | 0 | 37 |
| **Negative** | 7 | 226 | 233 |
| **Invalid** | 1 | 4 | 5 |
| **Total** | 45 | 230 | 275 |

CTA = clinical trial assay; F1CDx = FoundationOne^®^CDx; *NTRK* = neurotrophic receptor tyrosine kinase.

The local CTA inferred NTRK3 gene fusions were considered fusion positive.
**Table S24. Concordance between F1CDx and local CTA methods for detection of *NTRK* gene fusions based on the local CTA results**

| **Measure of agreement** | **Excluding CDx Invalid Results** | | **Including CDx Invalid Results** | |
| --- | --- | --- | --- | --- |
|  | **% Agreement (N)** | **95% CI^a^** | **% Agreement (N)** | **95% CI^a^** |
| PPA | 84.1% (37/44) | 69.9%, 93.4% | 82.2% (37/45) | 67.9%, 92.0% |
| NPA | 100.0% (226/226) | 98.4%, 100.0% | 98.3% (226/230) | 95.6%, 99.5% |
| OPA | 97.4% (263/270) | 94.7%, 99.0% | 95.6% (263/275) | 92.5%, 97.7% |

CDx = companion diagnostic; CI = confidence interval; CTA = clinical trial assay; NPA = negative percent agreement; OPA = overall precent agreement; PPA = positive percent agreement.

^a^ The 95% CI was calculated using the Clopper-Pearson exact method.

**Table S25. Agreement between F1CDx and CTA based on CTA results in Cohorts 4 and 5b for samples that met the F1CDx standard sample requirements for *MET* exon 14 skipping mutation**

| **Measure of agreement** | **Without F1CDx Invalid** | | **With F1CDx Invalid** | |
| --- | --- | --- | --- | --- |
|  | **Percent agreement, % (n/N)** | **95% CI** | **Percent agreement, % (n/N)** | **95% CI** |
| **Cohort 4** | | | | |
| PPA | 96.8 (30/ 31) | 83.3, 99.9 | 96.8 (30/ 31) | 83.3, 99.9 |
| NPA | 100 (84/ 84) | 95.7, 100 | 100 (84/ 84) | 95.7, 100 |
| OPA | 99.1 (114/115) | 95.3, 100 | 99.1 (114/115) | 95.3, 100 |
| **Cohort 5** | | | | |
| PPA | 100 (14/ 14) | 76.8, 100 | 100 (14/ 14) | 76.8, 100 |
| NPA | 100 (37/ 37) | 90.5, 100 | 100 (37/ 37) | 90.5, 100 |
| OPA | 100 (51/ 51) | 93.0, 100 | 100 (51/ 51) | 93.0, 100 |

CI = confidence interval; CTA = clinical trial assay; F1CDx = FoundationOne^®^CDx; NPA = negative percent agreement; OPA = overall percent agreement; PPA = positive percent agreement.

Table S26. Contingency table comparing F1CDx with the FoundationOne (F1) assay (CTA) on the detection of *FGFR* rearrangements for pemigatinib

|  | | **F1^a^ Assay (CTA)** | | | **Predictive value (95% CI)** |
| --- | --- | --- | --- | --- | --- |
|  |  | ***FGFR2***  **RE-positive** | ***FGFR2***  **RE-negative** | **Total** |  |
| **F1CDx** | *FGFR2*  RE-positive | 84^a^ | 0 | 84 | PPV: 100.00%  (73.14%, 100.00%) |
|  | *FGFR2*  RE-negative | 0 | 97 | 97 | NPV: 100.00%  (99.53%, 100.00%) |
|  | Total | 84 | 97 | 181 |  |
| Agreement (95% CI) | | PPA: 100.00%  (95.70%, 100.00%) | NPA: 100.00%  (96.27%, 100.00%) |  | OPA: 100.00%  (97.98%, 100.00%) |

CI = confidence interval; CTA = clinical trial assay; F1CDx = FoundationOne^®^CDx; NPA = negative percent agreement; OPA = overall percent agreement; PPA = positive percent agreement; RE = rearrangements.

^a^ One (1) sample (TRF322670) was enrolled by the FoundationOne Heme assay and was analyzed as an FoundationOne result for the concordance analysis.

Table S27. Concordance table comparing F1CDx with the CTA on the detection of *FGFR2* rearrangements for infigratinib

|  | | **CTA Assay** | | | **Predictive value** |
| --- | --- | --- | --- | --- | --- |
|  |  | ***FGFR2***  **RE-positive** | ***FGFR2***  **RE-negative** | **Total** |  |
| **F1CDx** | *FGFR2*  RE-positive | 58 | 0 | 58 | PPV: 100.00% |
|  | *FGFR2*  RE-negative | 2 | 102 | 104 | NPV: 99.65% |
|  | F1CDx-unevaluable | 39 | 3 | 42 |  |
|  | *FGFR2* F1CDx RE- positive | 9^a^ | 0 | 9 |  |
|  | Total | 108 | 105 | 213 |  |
| Agreement (95% CI) | | PPA: 96.67%  (88.64%, 99.08%) | NPA: 100.00%  (96.37%, 100.00%) |  |  |

CI = confidence interval; CTA = clinical trial assay; F1CDx = FoundationOne^®^CDx; NPA = negative percent agreement; OPA = overall percent agreement; PPA = positive percent agreement; RE = rearrangements.

^a^ Nine (9) *FGFR2* rearrangement positive samples were not included in the concordance analysis because they were enrolled by F1CDx. They are not included in the denominator in calculating PPA and NPA.


**Table S28. Agreement between F1CDx and CTA based on CTA1 and CTA2 results for *PIK3CA* alterations**

| **Measure of agreement** | **Without F1CDx Invalid** | | **With F1CDx Invalid** | |
| --- | --- | --- | --- | --- |
|  | **Percent agreement, % (n/N)** | **95% CI^a^** | **Percent agreement, % (n/N)** | **95% CI^a^** |
| **CTA1** | | | | |
| PPA | 93.8% (106/113) | 87.7%, 97.5% | 93.0% (106/114) | 86.6%, 96.9% |
| NPA | 98.8% (159/161) | 95.6%, 99.8% | 95.8% (159/166) | 91.5%, 98.3% |
| OPA | 96.7% (265/274) | 93.9%, 98.5% | 94.6% (265/280) | 91.3%, 97.0% |
| **CTA2** | | | | |
| PPA | 91.6% (197/215) | 87.1%, 95.0% | 90.4% (197/218) | 85.7%, 93.9% |
| NPA | 98.8% (162/164) | 95.7%, 99.9% | 97.0% (162/167) | 93.2%, 99.0% |
| OPA | 94.7% (359/379) | 92.0%, 96.7% | 93.2% (359/385) | 90.3%, 95.5% |

CI = confidence interval; CTA = clinical trial assay; F1CDx = FoundationOne^®^CDx; NPA = negative percent agreement; OPA = overall percent agreement; PPA = positive percent agreement.

Samples not tested are excluded from the analysis. Samples tested on deviation are excluded from the analysis.

^a^ The 95% CI calculated using the Clopper-Pearson Exact method.

**Table S29. Investigator-assessed PFS using F1CDx**

| **Cohort** | **Hazard Ratio**  **Rucaparib vs Placebo** | **Number of Patients** | **Median invPFS (months)** | | **95% CI** |
| --- | --- | --- | --- | --- | --- |
| ITT | 0.365 | 375 | 10.8 | Rucaparib | 8.3, 11.4 |
|  | *P*<0.0001 |  |  |  |  |
|  | 95% CI: 0.295, 0.451 | 189 | 5.4 | Placebo | 5.3, 5.5 |
| All populations assessable by FMI assays | 0.377 | 345 | 10.4 | Rucaparib | 8.3, 11.1 |
|  | *P*<0.0001 |  |  |  |  |
|  | 95% CI: 0.302, 0.469 | 173 | 5.4 | Placebo | 5.3, 5.5 |
| HRD+ | 0.302 | 215 | 13.6 | Rucaparib | 10.9, 17.1 |
|  | *P*<0.0001 |  |  |  |  |
|  | 95% CI: 0.224, 0.406 | 110 | 5.4 | Placebo | 5.1, 5.6 |
| *tBRCA*+ | 0.240 | 124 | 16.6 | Rucaparib | 11.1, 22.9 |
|  | *P*<0.0001 |  |  |  |  |
|  | 95% CI: 0.159, 0.364 | 63 | 5.4 | Placebo | 4.9, 7.1 |
| *tBRCA*-LOH+ | 0.354 | 91 | 9.7 | Rucaparib | 8.2, 13.8 |
|  | *P*<0.0001 |  |  |  |  |
|  | 95% CI: 0.226, 0.554 | 47 | 5.4 | Placebo | 2.9, 5.6 |
| *tBRCA*-LOH unknown | 0.176 | 16 | 8.3 | Rucaparib | 5.3, 24.7 |
|  | *P*=0.0069 |  |  |  |  |
|  | 95% CI: 0.044, 0.711 | 8 | 4.1 | Placebo | 2.3, 8.2 |
| *tBRCA*-LOH- | 0.620 | 114 | 6.3 | Rucaparib | 5.4, 8.3 |
|  | *P*=0.0086 |  |  |  |  |
|  | 95% CI: 0.429, 0.895 | 55 | 5.4 | Placebo | 4.1, 5.6 |

CI = confidence interval; F1CDx = FoundationOne^®^CDx; FMI = Foundation Medicine, Inc.; HRD = homologous recombination deficiency; invPFS = investigator assessed progression-free survival; ITT = intent-to-treat; LOH = loss of heterozygosity; *tBRCA =* tumor *BRCA* status.

**Table S30. Summary of clinical efficacy results for HRRm patients as determined by F1CDx in Cohort A**

|  | **Full analysis set** | | **F1CDx confirmed subgroup** | |
| --- | --- | --- | --- | --- |
|  | Olaparib (N=84) | NHA (N=43) | Olaparib (N=84) | NHA (N=43) |
| ORR, n (%)  (95% CI) | 28 (33)  (23, 45) | 1 (2)  (0, 12) | 27 (33)  (23. 44) | 1 (2)  (0, 12) |
| P-value | <0.0001 | | <0.0001 | |
|  | Olaparib (N=162) | NHA (N=83) | Olaparib (N=157) | NHA (N=83) |
| rPFS | | | | |
| Events^a^, n (%) | 106 (65) | 68 (82) | 101 (64) | 68 (82) |
| Median rPFS, months (95% CI) | 7.4  (6.2, 9.3) | 3.6  (1.9, 3.7) | 7.4  (6.9, 9.3) | 3.6  (1.9, 3.7) |
| HR (95% CI)b | 0.34 (0.25, 0.47) | | 0.33 (0.24, 0.46) | |
| Two-sided *P*-value^c^ | <0.0001 | | <0.0001 | |
| OS | | | | |
| Events, n (%) | 54 (33) | 39 (47) | 51 (32) | 39 (47) |
| Median OS, months (95% CI) | 18.5  (17.2, NR) | 15.1  (11.3, 19.1) | 18.5  (17.2, NR) | 15.1  (11.3, 19.1) |
| HR (95% CI) | 0.64 (0.43, 0.97) | | 0.62 (0.41, 0.95) | |
| Two-sided *P*-value | 0.0173 | | 0.0158 | |

BICR = blinded independent central review; CI = confidence interval; FAS = full analysis set; HR = hazard ratio; NHA = new hormonal agent; OS = overall survival; PCWG-3 = Prostate Cancer Working Group 3; RECIST = Response Evaluation Criteria in Solid Tumors; rPFS = radiological progression-free survival.

^a^ Progression, as assessed by BICR, was defined by RECIST 1.1 and/or PCWG-3 or death (by any cause in the absence of progression) regardless of whether the patient withdrew from randomized therapy or received another anticancer therapy prior to

progression.

^b^ The HR and CI were calculated using a Cox proportional hazards model adjusted for the variables selected in the primary pooling strategy (prior taxane use and measurable disease in Cohort A).

^c^ The analysis was performed using the log-rank test stratified by the variables selected in the primary pooling strategy (prior taxane use and measurable disease in Cohort A) using the Breslow method for handling ties.

**Table S31. Representation of disease ontology in the F1CDx orthogonal NGS concordance validation**

| **Disease Ontology** | **Number of Samples Evaluated** |
| --- | --- |
| Adrenal gland cortical carcinoma | 5 |
| Anus squamous cell carcinoma | 5 |
| Appendix adenocarcinoma | 7 |
| Bladder urothelial (transitional cell) carcinoma | 10 |
| Brain glioblastoma (GBM) | 6 |
| Brain gliosarcoma | 1 |
| Breast carcinoma (NOS) | 1 |
| Breast invasive ductal carcinoma (IDC) | 3 |
| Breast invasive lobular carcinoma (ILC) | 1 |
| Cervix adenocarcinoma | 7 |
| Colon adenocarcinoma (CRC) | 6 |
| Duodenum adenocarcinoma | 7 |
| Fallopian tube serous carcinoma | 4 |
| Gallbladder adenocarcinoma | 7 |
| Gastroesophageal junction adenocarcinoma | 1 |
| Head and neck squamous cell carcinoma (HNSCC) | 6 |
| Kidney renal cell carcinoma (NOS) | 7 |
| Liver hepatocellular carcinoma (HCC) | 7 |
| Lung adenocarcinoma | 3 |
| Lung large cell carcinoma | 1 |
| Lung non-small cell lung carcinoma (NOS) | 2 |
| Lung squamous cell carcinoma (SCC) | 1 |
| Nasopharynx and paranasal sinuses adenocarcinoma | 1 |
| Nasopharynx and paranasal sinuses squamous cell carcinoma | 2 |
| Nasopharynx and paranasal sinuses undifferentiated carcinoma | 4 |
| Ovary serous carcinoma | 7 |
| Pancreas ductal adenocarcinoma | 6 |
| Penis squamous cell carcinoma (SCC) | 6 |
| Pleura mesothelioma | 7 |
| Prostate acinar adenocarcinoma | 6 |
| Salivary gland carcinoma (NOS) | 3 |
| Skin adnexal carcinoma | 1 |
| Skin melanoma | 4 |
| Small intestine adenocarcinoma | 7 |
| Stomach adenocarcinoma (NOS) | 3 |
| Stomach adenocarcinoma diffuse type | 4 |
| Thymus carcinoma (NOS) | 4 |
| Thyroid follicular carcinoma | 1 |
| Thyroid papillary carcinoma | 5 |
| Ureter urothelial carcinoma | 4 |
| Urethra urothelial carcinoma | 2 |
| Uterus endometrial adenocarcinoma (NOS) | 2 |
| Uterus endometrial adenocarcinoma clear cell | 1 |
| Uterus endometrial adenocarcinoma endometrioid | 4 |
| Vagina squamous cell carcinoma (SCC) | 4 |
| Vulva squamous cell carcinoma (SCC) | 2 |

NOS = not otherwise specified.

**Table S32. Tumor profiling LoD for SUBs (MAF), INDELs (MAF), CNAs (tumor purity) and rearrangements (tumor purity) calculated using hit rate approach**

| **Variant Type** | **Variant Size** | **Genomic Context** | **N** | **Min** | **1^st^ Quartile** | **Mean** | **Med** | **3^rd^ Quartile** | **Max** |
| --- | --- | --- | --- | --- | --- | --- | --- | --- | --- |
| Substitution | - | - | 200 (198^a^) | 1.8% | 7.1% | 7.9% | 8.1% | 9.3%  (9.2%^a^) | 15.2% |
| Short Insertion | 1bp | - | 4 | 4.5% | 6.4% | 7.0% | 7.2% | 7.8% | 9.2% |
| Short Insertion | 1-2bp | Homopolymer Repeats | 2 | 11.6% | 12.1% | 12.6% | 12.6% | 13.1% | 13.6% |
| Short Insertion | 1-2bp | Dinucleotide Repeats | 1 | 9.3% | 9.3% | 9.3% | 9.3% | 9.3% | 9.3% |
| Short Insertion | 3-5bp | - | 2 | 6.0% | 6.2% | 6.4% | 6.4% | 6.6% | 6.8% |
| Short Insertion | >5bp | - | 2 | 6.0% | 6.7% | 7.4% | 7.4% | 8.2% | 8.9% |
| Short Deletion | 1bp | - | 3 | 5.4% | 6.8% | 7.6% | 8.2% | 8.7% | 9.3% |
| Short Deletion | 1-2bp | Homopolymer Repeats | 15 | 10.0% | 11.5% | 14.6% | 13.7% | 17.1% | 20.4% |
| Short Deletion | 1-2bp | Dinucleotide Repeats | 1 | 6.5% | 6.5% | 6.5% | 6.5% | 6.5% | 6.5% |
| Short Deletion | 3-5bp | - | 4 | 7.5% | 8.1% | 8.8% | 8.7% | 9.4% | 10.2% |
| Short Deletion | >5bp | - | 3 | 7.1% | 7.5% | 8.1% | 7.9% | 8.6% | 9.3% |
| Amplification | - | - | 15 | 9.6% | 10.6% | 21.7% | 18.5% | 22.4% | 58.3%^b^ |
| Homozygous deletion | - | - | 3 | 33.4% | 33.4% | 33.4% | 33.4% | 33.4% | 33.4% |
| Rearrangement | - | - | 3 | 9.2% | 9.2% | 11.1% | 9.2% | 12.1% | 14.9% |

Max = maximum; Med = median; Min = minimum.

^a^ Two TERT promoter substitutions removed.

^b^ VUS alteration at calling threshold

**Table S33. Summary of representative LoD for F1CDx platform (SVs)**

| **Variant Category** | **Subcategory** | **N** | **Range LoD^a^**  **Allele Fraction (%)** |
| --- | --- | --- | --- |
| SUBs | Known^b^ | 21^c^ | 1.8-7.9^c^ |
|  | Other^d^ | 166 | 5.9-11.8 |
| INDELs at non-homopolymer context, including insertions up to 42bp and deletions up to 276bp | Known | 3 | 4.5-6.5 |
|  | Other | 17 | 6.0-10.2 |
| INDELs at homopolymer context | 5bp repeat | 8 | 10.0-12.2 |
|  | 6bp repeat | 2 | 13.6-13.7 |
|  | 7bp repeat | 4 | 16.3-20.4 |
|  | 8bp repeat | 3 | 17.0-20.0 |

bp = base pair; INDEL = insertion/deletion; LoD = limit of detection; SUB – base substitution; SV = short variants; VUS = variants of unknown significance.

^a^ LoD calculations for the platform variants were based on the hit rate approach for variants with less than three levels with hit rate between 10% and 90% and probit approach for variants with at least three levels with hit rate between 10% and 90%. LoD from the hit rate approach is defined as the lowest level with 95% hit rate (worst scenario).

^b^ Alterations classified as” known” are defined as those that are listed in COSMIC

^c^ Data includes an alteration in the *TERT* promoter, 124C>T (LoD of 7.9%). *TERT* is the only promoter region interrogated and is highly enriched for repetitive context of poly-Gs, not present in coding regions.

^d^ Alterations classified as “other” include truncating events in tumor suppressor genes (splice, frameshift and nonsense) as well as variants that appear in hot-spot locations but do not have a specific COSMIC association, or are considered VUS due to lack of reported evidence and conclusive change in function.

**Table S34. Summary of representative analytical sensitivity for tumor purity for F1CDx platform alterations (copy number variants and rearrangements)**

| **Variant Category** | **N** | **Range**  **Tumor Purity (%)^a^** |
| --- | --- | --- |
| Copy Number Amplifications (CN>10)  Copy Number Amplifications (6≤CN≤10) | 8  7 | 9.6%-18.5%  19.5%-58.3%^b^ |
| Copy Number: Homozygous Deletions | 3 | 33.4%-33.4% |
| Genomic Rearrangements | 3 | 9.2%-14.9% |

CN = copy number; LoD = limit of detection; VUS = variants of unknown significance.

**^a^** Sensitivity calculations for the platform variants were based on the hit rate approach for variants with less than three levels with hit rate between 10% and 90% and probit approach for variants with at least three levels with hit rate between 10% and 90%. LoD from the hit rate approach is defined as the lowest level with 95% hit rate (worst scenario).

^b^ Max represents VUS alteration at calling threshold.

**Table S35. LoD summary statistics for TMB-H using component variants by MAF**

| **Variant Type** | **N** | **Minimum**  **(MAF)** | **1^st^ Quartile (MAF)** | **Median (MAF)** | **Mean (MAF)** | **3^rd^ Quartile (MAF)** | **Maximum (MAF)** |
| --- | --- | --- | --- | --- | --- | --- | --- |
| SUBs | 198 | 5.94% | 7.33% | 8.04% | 8.52% | 9.19% | 15.00% |
| INDELs | 7 | 6.32% | 7.08% | 8.94% | 10.28% | 11.74% | 19.05% |
| **Overall** | 205 | 5.94% | 7.32% | 8.05% | 8.58% | 9.24% | 19.05% |

INDEL = insertion/deletion; LoD = limit of detection; MAF = mutation allele frequency; SUB = substitution; TMB = tumor mutational burden.

Table S36. LoD Summary for MSI-H calling using tumor purity

| **Source Sample** | **Disease Ontology** | **MSI-H LoD by Hit Rate (%Tumor Purity)** |
| --- | --- | --- |
| 1 | Colon adenocarcinoma (CRC) | 8.33% |
| 2 | Colon adenocarcinoma (CRC) | 8.25% |
| 3 | Colon adenocarcinoma (CRC) | 12.01% |
| 4 | Colon adenocarcinoma (CRC) | 15.67% |
| 5 | Uterus endometrial adenocarcinoma (NOS) | 8.85% |

CRC = colorectal cancer; LoD = limit of detection; MSI = microsatellite instability; MSI-H = microsatellite instability-high; NOS = not otherwise specified.

**Table S37. Positive and negative call rates per sample for platform variants (N=717)**

| **Alteration type(s) assessed** | **Positive call rate** | **Exact 95% CI** | | **Negative call rate** | **Exact 95% CI** | |
| --- | --- | --- | --- | --- | --- | --- |
|  |  | **Lower** | **Upper** |  | **Lower** | **Upper** |
| CNA/RE/SUB | 100.00% | 99.40% | 100.00% | 99.98% | 99.95% | 99.99% |
| CNA/ SUB/INDEL | 99.37% | 98.38% | 99.83% | 99.96% | 99.92% | 99.98% |
| SUB/INDEL | 100.00% | 99.10% | 100.00% | 99.97% | 99.95% | 99.99% |
| CNA/ SUB/INDEL | 97.84% | 96.89% | 98.56% | 99.84% | 99.78% | 99.89% |
| SUB/INDEL | 99.81% | 98.94% | 100.00% | 99.98% | 99.95% | 99.99% |
| SUB/INDEL | 99.60% | 97.81% | 99.99% | 99.94% | 99.90% | 99.97% |
| CNA/ SUB/INDEL | 98.33% | 97.11% | 99.14% | 99.98% | 99.96% | 100.00% |
| SUB/INDEL | 100.00% | 99.83% | 100.00% | 99.97% | 99.94% | 99.99% |
| CNA/ SUB/INDEL | 100.00% | 99.32% | 100.00% | 99.98% | 99.96% | 100.00% |
| RE/ SUB/INDEL | 96.46% | 94.14% | 98.05% | 99.96% | 99.92% | 99.98% |
| CNA/ SUB | 98.67% | 97.27% | 99.46% | 99.98% | 99.96% | 100.00% |
| CNA/RE/SUB/INDEL | 96.27% | 95.39% | 97.02% | 99.87% | 99.82% | 99.91% |
| RE/SUB/INDEL | 98.23% | 97.48% | 98.80% | 99.66% | 99.58% | 99.73% |
| CNA/ SUB/INDEL | 98.32% | 97.57% | 98.89% | 99.92% | 99.88% | 99.95% |
| SUB/INDEL | 99.30% | 98.90% | 99.58% | 99.90% | 99.86% | 99.94% |
| CNA/RE/SUB/INDEL | 85.42% | 82.27% | 88.20% | 99.89% | 99.84% | 99.93% |
| RE/SUB/INDEL | 97.75% | 96.42% | 98.68% | 99.98% | 99.95% | 99.99% |
| RE/SUB/INDEL | 95.30% | 92.97% | 97.03% | 99.96% | 99.93% | 99.98% |
| CNA/RE/SUB/INDEL | 100.00% | 98.31% | 100.00% | 99.89% | 99.84% | 99.93% |
| CNA/RE/SUB/INDEL | 100.00% | 99.25% | 100.00% | 99.96% | 99.93% | 99.98% |
| CNA /SUB | 96.83% | 94.90% | 98.17% | 99.94% | 99.90% | 99.97% |
| CNA/RE/SUB/INDEL | 95.97% | 94.06% | 97.40% | 99.98% | 99.96% | 100.00% |
| CNA/ SUB/INDEL | 100.00% | 99.42% | 100.00% | 99.93% | 99.89% | 99.96% |
| CNA/RE/SUB/INDEL | 100.00% | 99.30% | 100.00% | 99.95% | 99.91% | 99.97% |
| RE/SUB | 100.00% | 99.05% | 100.00% | 100.00% | 99.98% | 100.00% |
| CNA /SUB | 96.99% | 95.39% | 98.15% | 99.84% | 99.79% | 99.89% |
| CNA/RE/SUB/INDEL | 100.00% | 98.95% | 100.00% | 99.93% | 99.89% | 99.96% |
| CNA/RE/SUB/INDEL | 99.80% | 99.29% | 99.98% | 99.98% | 99.96% | 100.00% |

CNA = copy number alteration; INDEL = insertion/deletion; RE = rearrangement; SUB = substitution.

**Table S38. Initial evidence of actionability of FMI CGP from selected pan- or multi-tumor studies (prospective or retrospective) enrolling patients from 2012−2017 in academic and community settings, collectively including 1,874 patients with FMI results prior to availability of F1CDx**

| **Actionability** | **Percent of patients (range of data from studies including each type of actionability data)** | **Number of studies providing data** |
| --- | --- | --- |
| Percent of patients with actionable alterations^a^ | 52-99% | 8 [4, 41, 93, 95-97, 100, 101] |
| Percent of patients pursuing Genomically Informed Therapy (GIT)^b^ | 16-49% | 10 [4, 41, 94-101] |
| Reasons for not pursuing GIT at time of study | **Treatment Factors:** ineligibility for clinical trial, contraindication, no access to therapy, using results for future line of therapy, selected standard therapy  **Disease Factors:** stable disease, no evidence of disease, clinical deterioration, death  **Physician or patient preference** | 11 [4, 41, 93-101] |

CGP = comprehensive genomic profiling; F1CDx = FoundationOne^®^CDx; FDA = Food and Drug Administration; GIT = genomically informed therapy; MSI = microsatellite instability-high; TMB = tumor mutational burden.

^a^ Definitions of actionable alterations generally included a genomic alteration or biomarker that can be targeted* with an FDA-approved therapy† (on-label or off-label), or an investigational agent through a clinical trial.

^b^ GIT: systemic therapy with an agent that targets an actionable alteration (or a pathway component of an actionable alteration) or that targets another tumor biomarker (e.g., MSI, TMB). In five of seven studies for which the number of prior lines of treatment was reported CGP was used to identify 3^rd^ or greater line of therapy in at least a majority of patients, therefore multiple publications mentioned the need to test earlier to maximize the benefit of CGP.

**Table S39. Clinical utility of CGP**

| **Author/year** | **Study design** | **Clinical impact** | | |
| --- | --- | --- | --- | --- |
|  |  | **Populations compared** | **Outcome measure^a^** | ***P*-value** |
| **Pan-tumor** | | | | |
| **Kato 2018^63^** | Prospective study of the utility of tissue and liquid CGP in patients with rare cancers (n=40) | Matched targeted therapy (n=12) | PFS: 19.7 months | *P*=0.008 |
|  |  | Previous unmatched therapy (n=12) | PFS: 3.5 months |  |
| **Schwaederle 2016^74^** | Retrospective study of the utility of CGP to match patient with advanced solid malignancies to a therapy (n=347) | Matched therapy (n=87) | DCR^b^: 34.5% | *P*≤0.02 |
|  |  | Unmatched therapy (n=93) | DCR^b^: 16.1% |  |
|  |  | Matched therapy (n=87) | PFS: 4.0 months | *P*=0.039 |
|  |  | Unmatched therapy (n=93) | PFS: 3.0 months |  |
| **Wheler 2016^67^** | Single-arm, nonrandomized study to prospectively investigate the clinical utility of CGP in patients with advanced malignancies (N=500) | Matched therapy (n=122) | DCR: 19% | *P*=0.61 |
|  |  | Unmatched therapy (n=66) | DCR: 8% |  |
|  |  | Matched therapy (n=122) | TTF: 2.7 months | *P*=0.001 |
|  |  | Unmatched therapy (n=66) | TTF: 1.9 months |  |
|  |  | Matched therapy (n=122) | OS: 9.3 months | *P*=0.087 |
|  |  | Unmatched therapy (n=66) | OS: 7.2 months |  |
| **Sicklick 2019^66^**  **I-PREDICT** | Prospective navigation trial at 2 centers using tissue-based CGP to match patients to therapies based on a matching score^c^ (n=73) | High matching score (n=28) | PFS: 6.5 months | *P=*0.046 |
|  |  | Low matching score (n=55) | PFS: 3.1 months |  |
| **Breast** | | | | |
| **Ganesan 2014^77^** | Retrospective study of consecutive patients with advanced or metastatic triple-negative metastatic breast cancer (N=106) treated in a phase 1 clinic | Matched therapy (n=90) | PFS: 6.4 months | *P=0.001* |
|  |  | Unmatched therapy (n=16) | PFS: 1.9 months |  |
| **Pancreatic** | | | | |
| **Pishvaian 2018^75^** | Prospective program (Know Your Tumor) using CGP to determine matched therapy in patients with pancreatic cancer (n=640) | Matched therapy (n=17) | PFS: 4.1 months | *P*=0.03 |
|  |  | Unmatched therapy (n=18) | PFS: 1.9 months |  |
| **Pishvaian 2020^78^** | Prospective program (Know Your Tumor) using CGP to determine matched therapy in patients with pancreatic cancer (n=1,856) | Matched therapy (n=46) | OS: 2.58 years | *P*=0.0004 |
|  |  | Unmatched therapy (n=143) | OS: 1.51 years |  |
| **NSCLC** | | | |  |
| **Singal 2019^79^** | A retrospective study to determine the clinical utility of a clinico-genomic database (using CGP) in patients with NSCLC (n=4,064) | Patients with driver alteration treated with targeted therapy (n=575) | OS: 18.6 months | *P*<0.001 |
|  |  | Patients with driver alteration not treated with targeted therapy (n=560) | OS: 11.4 months |  |
| **Madison 2020^97^** | A retrospective, real-world study to determine clinical outcomes for NSCLC patients following CGP with liquid biopsy or tissue biopsy (n=6,491) | Patients with genomic alteration matched to targeted therapy (n=287) | rwPFS: 9.4 months | *P*=0.022 |
|  |  | Patients with genomic alteration not treated with targeted therapy (n=130) | rwPFS: 6.9 months |  |
|  |  | Patients with genomic alteration matched to targeted therapy (n=262) | OS: 26.7 months | *P*=0.035 |
|  |  | Patients with genomic alteration not treated with targeted therapy (n=130) | OS: 17.9 months |  |

CGP = comprehensive genomic profiling; DCR = disease control rate; NSCLC = non-small cell lung cancer; ORR = overall response rate; OS = overall survival; PFS = progression-free survival; rwPFS = real-world progression-free survival; TTF = time to treatment failure.

^a^ PFS, TTF, and OS as presented above are all measured as medians.

^b^ DCR is the percentage of patients achieving a complete response, partial response, or stable disease for ≥6 months.

^c^ A “matching score” score system was then utilized for each patient. Blinded to patient outcomes, the investigators calculated the total number of molecular alterations matched to the drugs administered and divided that number by the total number of characterized genomic aberrations.

**Table S40. Frequency of F1CDx reports with actionable alterations potential therapeutic implications by disease group and definition of actionability: number of samples by disease group; therapy options available within the Tumor Type indicated; therapy options available in Tumor Types other than the assigned indication; disease groups with clinical trial options; and, companion/complementary diagnostics**

| **Disease Group** | **Total Number of Samples** | **Therapy Options Available In Tumor Type** | **Therapy Options Available Outside of Tumor Type** | **Clinical Trial Available** | **Companion Diagnostic Available** |
| --- | --- | --- | --- | --- | --- |
| Lung non-small cell lung carcinoma (NSCLC) | 36179 | 25674 | 28729 | 34480 | 18177 |
| Colorectal (CRC) | 24608 | 10918 | 10554 | 21342 | 14696 |
| Breast | 20410 | 15632 | 15943 | 18547 | 6292 |
| Pancreas | 12902 | 699 | 6380 | 12337 | 257 |
| Ovary | 11891 | 3384 | 7299 | 10568 | 5301 |
| Prostate | 9745 | 2298 | 5793 | 7935 | 2693 |
| Unknown primary carcinoma (CUP) | 8887 | 1307 | 6400 | 7746 | 1737 |
| Endometrial | 6398 | 1279 | 5493 | 5928 | 1379 |
| Esophagus | 5789 | 1399 | 4126 | 5051 | 556 |
| Melanoma | 5602 | 5048 | 4709 | 5427 | 4446 |
| Bladder | 4957 | 2785 | 4068 | 4601 | 1751 |
| Glioma | 4393 | 49 | 3761 | 4010 | 204 |
| Cholangiocarcinoma | 4208 | 275 | 2598 | 3475 | 505 |
| Stomach | 3382 | 770 | 2159 | 2733 | 340 |
| Head and neck | 3330 | 688 | 2168 | 2703 | 623 |
| Kidney | 3262 | 1731 | 1633 | 2375 | 230 |
| Small cell | 2288 | 434 | 1204 | 1763 | 809 |
| Cervix | 1867 | 304 | 1425 | 1572 | 380 |
| Thyroid | 1578 | 331 | 1354 | 1401 | 61 |
| Fallopian tube | 1335 | 386 | 772 | 1225 | 47 |
| Small intestine | 1295 | 134 | 953 | 1224 | 157 |
| Biliary | 1273 | 62 | 860 | 1081 | 91 |
| Uterus | 1024 | 79 | 801 | 929 | 82 |
| Liver | 891 | 65 | 322 | 650 | 46 |
| GIST | 856 | 748 | 796 | 766 | 7 |
| Skin | 830 | 472 | 676 | 734 | 549 |
| Appendix | 768 | 17 | 452 | 649 | 35 |
| Mesothelioma | 718 | 7 | 411 | 470 | 14 |
| Salivary gland | 706 | 55 | 472 | 532 | 97 |
| Anus | 682 | 81 | 455 | 511 | 121 |
| Peritoneum | 645 | 159 | 336 | 552 | 14 |
| Female genital | 606 | 61 | 410 | 462 | 92 |
| Adenoid cystic carcinoma | 606 | 4 | 175 | 288 | 3 |
| Urinary | 524 | 223 | 400 | 466 | 114 |
| Soft tissue sarcoma | 435 | 17 | 192 | 289 | 17 |
| Carcinoid | 414 | 36 | 111 | 179 | 14 |
| Adrenal gland | 277 | 9 | 70 | 164 | 21 |
| Germ cell | 252 | 9 | 103 | 161 | 17 |
| Leiomyosarcoma | 200 | 5 | 67 | 91 | 6 |
| Peripheral nervous system (PNS) | 187 | 5 | 70 | 88 | 5 |
| Male genital | 176 | 21 | 131 | 141 | 27 |
| Thymus | 163 | 11 | 58 | 72 | 13 |
| Thymus thymoma | 127 | 1 | 23 | 37 | 2 |
| Head and neck-neuro | 112 | 4 | 35 | 50 | 7 |
| Bone sarcoma | 100 | 3 | 30 | 46 | 3 |

Source: from over 504,000 FMI profiles consented for secondary research, 191,575 unique US patients tested with F1CDx from Jan. 14, 2018 through Mar. 31, 2021.
