# Supplementary material for: Clinical and analytical validation of FoundationOne®CDx, a comprehensive genomic profiling assay for solid tumors
Source: PLoS One. 2022 Mar 16;17(3):e0264138. doi: 10.1371/journal.pone.0264138 (PMC8926248; doi:10.1371/journal.pone.0264138)
Supplement: S2 Appendix — (DOCX) [file pone.0264138.s002.docx]

**S2 Supplementaary Appendix: Supplementary Methods**

**Device validation population for TMB-H in solid tumors.** For the clinical validation of TMB-H in solid tumors, the DV population was defined based on the F1CDx final sample specification. Of the 735 subjects with a valid TMB result, 272 subjects had samples that failed to meet the minimum test specifications: five participants did not pass preanalytical QC due to insufficient sample material for testing (n=1) or gender discordance (n=4); 156 did not pass pathology review based on F1CDx device specifications (i.e., >20% tumor nuclei and >0.6 mm^3^ of viable nucleated tissue); 40 did not meet the minimum extracted DNA criterion (≥55 ng); and 71 yielded invalid results after sequencing and reporting. Among the 735 patients with valid TMB results, 13 were enrolled <26 weeks before the data cut-off for IA10, two were not treated, and one did not have a TMB score; as a result, the DV population consisted of 719 patients with valid TMB scores. Among these 719 participants, 91 were TMB-H (≥10 mut/Mb) and 628 were non-TMB-H (<10 mut/Mb).

**Computational tumor purity.** Computational tumor purity is calculated by fitting the observed log-ratio and MAF data with statistical models that predict a genome-wide copy number profile, tumor ploidy, and tumor purity (i.e., computational tumor purity). The log-ratio profile is obtained by normalizing aligned tumor sequence reads by dividing read depth by that of a process-matched normal control, followed by a GC-content bias correction using Loess regression. The minor allele frequency profile is obtained from the heterozygous genome-wide SNPs. For platform-wide LoD assessment, the INDELs were grouped together (other than homopolymer repeat context) as they are similar in LoD characteristics. The INDELs ranged from 1 bp up to 42 bp INDELs up to 276 bp. INDELs at homopolymer repeat context had higher LoD, with a dependency on the length of the repeat context.

**Samples analyzed for the orthogonal concordance for the companion diagnostic claims**. The samples analyzed for orthogonal concordance included:

101 (53 positive, 48 negative by F1CDx) breast cancer samples were analyzed to determine concordance specific to *PIK3CA* base substitutions;

158 (26 positive, 132 negative by F1CDx) cholangiocarcinoma samples were analyzed to determine concordance to an externally validated laboratory developed test specific to *FGFR2* fusions and select rearrangements with additional samples to be completed in the post-market setting;

168 (50 positive, 118 negative by F1CDx) NSCLC samples were analyzed to determine concordance for detection of qualifying *MET* exon 14 base substitutions and INDELs;

230 (120 positive, 110 negative by F1CDx) samples were analyzed to determine concordance specific to HRR alterations (including SUBs, INDELs, rearrangements and homozygous deletions);

218 (76 TMB-H, 146 non-TMB-H by F1CDx) samples were analyzed to determine concordance with a CLIA validated WES assay for detection of TMB ≥10 mut/Mb; and

626 solid tumor samples were analyzed to determine concordance of *NTRK1/2/3* fusions. These included 588 (88 positive, 500 negative by F1CDx) samples where F1CDx served as the selection assay (subset 1) and 38 (15 positive, 23 negative by F1CDx) clinical trial samples where local CTAs served as the selection assay (subset 2).
